# Remote work – the new normal needs more research

**DOI:** 10.5271/sjweh.4213

**Published:** 2025-03-01

**Authors:** Annina Ropponen

One of the recent global crises has been the COVID-19 pandemic, drastically changing how we work in expert positions or otherwise do work that can be performed using mobile devices. This change has been seen in many studies since the onset of the pandemic. In the *Scandinavian Journal of Work, Environment & Health*, papers about remote work (alternative search terms telework, hybrid work) have increased from zero, before 2021, to four [see eg (1–4),]. However, the increase is even more clear in PubMed-indexed papers, where the number of articles was 1–10 per year from 1990 to 2019, 134 in 2020, and 346–470 per year in 2021–2023. However, despite this urgent interest in this topic (5, 6), we still have some knowledge gaps.

## The sweet child has many names

Although remote work has existed for decades (7), it has many names. As indicated above, terminology that has been used in the literature varies, such as telework, work from home, and hybrid work, where people work both remotely and at their employer’s premises. The European Foundation for the Improvement of Living and Working Conditions (Eurofound) has used the term telework, defining it as “a work arrangement in which work is performed outside a default place of work, normally the employer’s premises, by means of information and communication technologies (ICT). The characteristic features of telework are the use of computers and telecommunications to change the usual location of work, the frequency with which the worker is working outside the employer’s premises, and the number of places where workers work remotely (mobility)” (8). Thus, these terms capture a variety of definitions while measures have mainly relied on survey items. Although surveys can specify the timing, ie, “Have you worked remotely in the past week?” or the average of the past month, they are self-reported and thus prone to recall and reporting biases. On the other hand, earlier research indicates that sometimes workers work a part of a specific day from home and the other part from their employer’s premises, thus the same day can be both an on-site and a remote workday (9, 10). How are these differences reflected in the survey items or should they be monitored differently? Since the last decade, the use of employer-owned register data for studies of working hours has increased [eg (11–13),] and such data could enable investigation of place for work (ie, where) in addition to timing (when) (14, 15). Thus, longitudinal studies using employer’s register data for working hours and places for working are needed to deepen the knowledge about the various ways of combining remote work and work at the employer premises and the subsequent effects on employee health, well-being, and work performance.

## The COVID-19 pandemic time dominates research on remote work, but we do not know much about the post-pandemic worklife

Besides the measures of remote work, another aspect hampering the current knowledge is the fact that studies of remote work among knowledge workers have been focused on the preceding or early years of the pandemic (16–19) or used cross-sectional design and/or survey data (18, 20, 21) mainly focused on the psychosocial aspects of the work environment. Thus, studies combining a longitudinal design with register data are needed (14, 19). Various factors influenced remote work during the COVID-19 pandemic. Regulations and guidelines at global, national, and regional levels dictated remote work practices, particularly concerning infectious disease symptoms. Logistical factors—such as the availability of public transportation and workspace capacity at employer premises—also impacted whether employees intended to work remotely, had the opportunity to do so, or were even allowed to by their employers (22, 23). Furthermore, infrastructure issues, such as fast internet connectivity and affordability of housing with a workspace (ie, enablers for working from home remotely) influence the possibility and attractiveness of remote work (24–26). These infrastructure issues related to nations and their cities, and employers might play a role that has not yet been elaborated for the future of work.

## Regulations and monitoring are needed

Remote work challenges employee well-being as we have become aware via national legislation to restrict connectivity after work (27). However, such restrictions are rather few; in Europe only nine countries (Belgium, Croatia, France, Greece, Italy, Luxembourg, Portugal, Slovakia, and Spain) have legislation providing a right to disconnect. Although interest in disconnecting after work has existed for decades, the research seems to be characterized by similar weaknesses as studies of remote work in general: vague terminology, cross-sectional designs, and self-reported data as indicated by a recent mapping review (28). Still, the evidence is rather clear on the mental and physical health benefits of allowing employees to disconnect and recover (29). This might suggest that along with the high prevalence of remote work, studies and efforts should be invested in national and local regulations and longitudinal, high-quality studies using daily register data on working when and where to address the link to employee well-being, health, and productivity (30). Daily register data could be targeted to monitor working hours (14, 31) and computer use (32) and access control to premises or other intelligent workplace systems (33) that enable the monitoring of employees or wearable technology such as smartwatches or access tags (34) for research purposes. Thus, by deepening our understanding of remote work and detachment from work, we might improve workplace productivity and human resources.

## From the responsibility of the employers to the freedom of employees

Remote work is often done from home or a place owned by the employee (ie, vacation home). Sociodemographic factors such as income and educational attainment strongly determine home sizes and their locations (ie, whether they are in cities or more rural) (25). Given the fact that a home is a private place where individuals live alone or with others, employers do not have a say on the living or working conditions. This is a challenge from the occupational safety and health (OSH) perspective. Most of the OSH legislation dictates the role of the employer in safeguarding their employees from risks and hazards. In remote work, employees set their working conditions and work, some with state-of-the-art workstations with wide screens and separate keyboards and electronic tables, while others might not have any dedicated workspaces but work sitting on the sofa or at the kitchen table. In the short-term or occasionally, this might be fun. However, the long-term effects of poor physical working conditions or positions may negatively impact musculoskeletal organs, eye ergonomics, or cognitive functions (35, 36). This is especially concerning since young employees or those with lower socioeconomic status might be more vulnerable to these working conditions in their homes and have less economic incentives or knowledge of possibilities to adjust them. This aspect related to office ergonomics in remote work has been acknowledged in the literature (37), but further research and emphasis should be placed on avoiding any new pandemic of musculoskeletal or other health complaints due to working from home.

## The rise and shine of remote work

One may speculate if the increased rates of remote work will remain. If we think about the employer perspective, working both remotely and on-site has been, and will continue to be, challenged, and potentially adds costs as the use of premises varies a lot. Even though there are a lot of positive aspects of remote work from the employee perspective, some negative aspects have also been highlighted such as social isolation, lack of peer or supervisor support, and spillover of work to leisure time, all of which have increased due to remote work (5, 6, 18). These do not account for the fact that, in general, the possibility of employees having control over their work is an important factor that is strongly linked with well-being and health (38, 39). Therefore, even in the discussion about remote work, an important reminder is needed. Remote work is a form of flexible working that can be defined as having employment agreements that promote employees’ control over when, where, for how long, and how continuously the employee works (40–42). This form of employee-oriented flexibility refers to the employee’s entitlement to influence when and where they work and according to their individual needs and wishes. This contrasts with arrangements in which the employer determines the employees’ working times and location according to the company’s needs (company-based flexibility). Another important fact that has been missed in the current remote work discussion is the fact that flexibility helps people align their work commitments with their private lives. Besides, flexibility allows employees better opportunities to recover from the strain and effort associated with work, both during and outside work. In these mechanisms, the immediate impact of flexibility leads to a reduction in stress, thereby promoting better health (43, 44). Thus, I dare to claim that soon, within 1–5 years, we will not discuss remote work but rather flexibility. The discussion will lead to legislation and regulations enabling flexibility in a way that promotes the health and employee well-being while at the same time ensuring a safe and healthy work environment. Such legislation and regulations will exist locally, nationally, and internationally as such flexibility will also enable working across country borders.
